# Perception and Knowledge of Final-Year Veterinary Students About Exotic Pet Mammals

**DOI:** 10.3390/vetsci12030235

**Published:** 2025-03-03

**Authors:** Mario Ostović, Ivana Sabolek, Aneta Piplica, Ivona Žura Žaja, Sven Menčik, Željko Pavičić, Željka Mesić

**Affiliations:** 1 Department of Animal Hygiene, Behavior and Welfare, Faculty of Veterinary Medicine, University of Zagreb, 10000 Zagreb, Croatia; isabolek@vef.unizg.hr (I.S.); zpavicic@vef.unizg.hr (Ž.P.); 2 Department of Animal Breeding and Livestock Production, Faculty of Veterinary Medicine, University of Zagreb, 10000 Zagreb, Croatia; apiplica@vef.unizg.hr; 3 Department of Physiology and Radiobiology, Faculty of Veterinary Medicine, University of Zagreb, 10000 Zagreb, Croatia; izzaja@vef.unizg.hr; 4 Department of Marketing in Agriculture, Faculty of Agriculture, University of Zagreb, 10000 Zagreb, Croatia; zmesic@agr.hr

**Keywords:** veterinary medicine, survey, exotic pets, risk perception

## Abstract

The ever-increasing number and variety of exotic pets have imposed an inevitable challenge to veterinary profession, expecting the veterinarians to safeguard health and welfare of both animals and humans, as well as the environment. This study investigated perception of last-year veterinary students concerning welfare issues, health and safety of humans and other animals, along with environmental protection associated with keeping exotic pet mammals, and their self-reported knowledge of these pets. The sixth-year veterinary students in Croatia were surveyed in the 2019–2020 academic year, with a response rate of 82% (95 students). The questionnaire was focused on rodents, rabbits, and ferrets. Students generally agreed on the issues related to the welfare of these pets, with their responses yielding no significant between-mammal differences. There were no significant differences among particular mammals either in the issues other than animal welfare, with which they did not agree or were uncertain. Students considered themselves to have appropriate knowledge about nutrition, housing, health and behavior of these pets. Previous experience with exotic pets showed a substantial association with student perception of these animals. Study results suggest an adequate student perception of exotic pet mammal welfare, along with their appropriate self-reported knowledge of these pets, however, veterinarians-to-be are expected to think twice about the implications that exotic pet animals may have for humans, other animals, and eventually for the environment. Therefore, there is a need of additional student education in the field through intensification of their interaction with these animals, which especially refers to human health issues associated with these animals.

## Simple Summary

Nowadays, the role of veterinarians has assumed new dimensions. Veterinarians are expected to safeguard not only health and welfare of animals but also of humans and the environment. The aim of this study was to investigate perception of final-year veterinary students in Croatia about exotic pet mammals, i.e., rodents, rabbits, and ferrets concerning the welfare of these pets, health and safety of humans and other animals, and environmental protection, and their self-reported knowledge of these animals. Results of the questionnaire survey indicate an adequate student perception of welfare issues in these pets and they stated to have appropriate knowledge about their nutrition, housing, health and behavior; yet, other issues of concern related to the threat posed by these pets to health and safety of humans and other animals, and the environment require more attention by students. Study results also showed a substantial association between previous experience with exotic pets and student perception of these pets, suggesting intensification of their interaction by additional education on these animals.

## 1. Introduction

Non-traditional or exotic pets are increasingly popular worldwide [1,2]. Comparing the European Union (EU), United States (US) and Canada, the largest number of exotic pets, i.e., nearly 70 million including reptiles, birds and mammals, are kept in the EU [3]. Although exotic pets are commonly defined as animals that are either non-native to a region or non-domesticated [4], from the veterinary point of view any pet that is neither a dog nor a cat is considered to be an exotic pet [5,6].

Many exotic animal species have complex needs that make them unsuitable as pet animals and their welfare can be compromised in any step of the trade and keeping chain [4]. The welfare of exotic pet animals is frequently endangered by the lack of specific information on their care, inappropriate husbandry, and unrealistic owner expectations even in case of pet animals such as rabbits and small rodents, which are traditionally considered as suitable pets for children. The issue is additionally aggravated by owner failure to recognize the signs of poor pet health, along with the lack of specialist veterinary care and lack of owner willingness to use this care [2,7]. Recent studies demonstrated that 40% and 34% of exotic pet owners, respectively, never had asked for veterinary service [8,9].

Exotic pets are a significant source of zoonotic diseases [10,11]. Additional issues of concern arising from exotic pets are other animal health [12] and antimicrobial resistance associated with misuse or overuse of chemical therapeutics and prophylaxis [13], highlighting the need of a reasonable use of antibiotics in such pets [14]. Moreover, exotic pets are known to cause injuries including death incidents [15,16], which in some cases have led to stricter regulation on exotic animal ownership in addition to the risk of disease [3]. Furthermore, the growing demand for exotic pets is an increasing driver of the global wildlife trade and loss of biodiversity [17,18]. Taking animals from the wilderness reduces their population and threatens species, while exotic animals kept as pets and either released by their owners or escaped can invade and jeopardize the non-native ecosystems [4,19].

Therefore, there is a need of developing guidelines on responsible ownership of exotic pets and codes of practice in animal care, with implications for veterinary profession along with providing pet owners with required service and counselling [9,20]. The role of veterinarians is ever more significant and necessary to support not only health and welfare of exotic pets, but also of humans and the environment, imposing inevitable challenges in veterinary profession, which needs to implement crucial changes in educational and training programs [21].

Previous studies involving exotic non-mammal pets suggest deficient veterinary perception, knowledge and education on these pets, although birds received higher scores as compared to reptiles, amphibians or fish [22,23]. Certain differences in veterinary student perception also were found regarding different pet reptiles observed [24].

The aim of this study was to investigate perception of veterinary students about exot-ic pet mammals, i.e., issues related to welfare of these pets, health and safety of humans and other animals, and environmental protection, and their self-reported knowledge about these pets. The study also investigated the association of student socio-demographic characteristics with their perception and knowledge about these animals.

## 2. Materials and Methods

A total of 95 (82%) students enrolled in the final, sixth-year of the integrated undergraduate and graduate study program at the only Faculty of Veterinary Medicine in Croatia, University of Zagreb, completed the survey at the beginning of the 2019–2020 academic year winter term. The students received a paper questionnaire in the classroom. Prior to the survey, students were explained the aim of the study and its voluntary and anonymous design. Students had the required course on animal welfare in the first study year but the knowledge and skills related to exotic pets were also acquired in other required courses throughout the study, with several elective courses focused on exotic non-mammal pets.

The questionnaire had two sections (see Supplementary Materials). The first section questions provided information on the student socio-demographic profile including gender, age, environment where they grew up, secondary school, prior experience of owning or keeping (exotic) pets, and study track chosen (fifth study year). Exotic pet experience implied any animals other than dogs and cats. The second section aimed at investigating student perception and their self-reported knowledge about exotic pet mammals using 10 statements in the form of 5-point Likert scale questions (1—completely disagree, 2—disagree, 3—neither agree nor disagree, 4—agree, 5—completely agree) and Yes/No/I do not know questions, and an open-ended question. The questions were focused on rodents, rabbits (*Oryctolagus cuniculus*) and ferrets (*Mustela putorius furo*). Additional Yes/No/I do not know question referred to exotic pets in general. A pre-survey was performed in 10% of students at the study year and a revised questionnaire was used thereafter.

All data were analyzed with IBM SPSS Statistics v. 21.0 (IBM Corp., Armonk, NY, USA, 2012). Univariate descriptive statistics was employed to analyze frequency and distribution of data. Differences in student responses related to pet mammals and education areas were analyzed by Kruskal–Wallis test and Mann–Whitney U-test. ANOVA test and χ2-test were used to determine the association between socio-demographic parameters and student responses to Likert scale questions and Yes/No/I do not know questions. The level of statistical significance was set at *p* < 0.05.

## 3. Results

The majority of study students were female (74.7%), aged ≥24 years (80%), grown up in urban environment (71.6%), with high school education (94.7%) and experience in owning or keeping pets (96.8%) including exotic pets (53.7%), and preferred career in pet medicine (49.5%) (Table 1). Concerning exotic pet animals owned or kept, students mostly reported on fish (25.7%), followed by birds (21.2%), rodents (18.6%), reptiles (15.9%) and rabbits (10.6%), while ferrets, hedgehogs, amphibians and invertebrates together accounted for 8% of owned or kept animals.

Students agreed that rodents, rabbits and ferrets were capable of thinking and feeling emotions, with the role of biological functioning, emotional states and natural living being important for their welfare. Students also agreed that welfare of these pets was compromised, ranking rodents and rabbits as significantly more suitable (*p* < 0.05) as pet animals in comparison with ferrets. They neither agreed nor disagreed that ferrets should be kept as pets. Students expressed the lowest level of agreement with the statement that owners were adequately informed on rodents and rabbits and their needs prior to getting them as pets; students neither agreed nor disagreed about the same issue related to ferrets, yielding no significant between-mammal differences (Table 2).

Concerning the most important issue related to the welfare of exotic pet mammals, the largest proportion of study students reported their inappropriate accommodation (36.7%), followed by the lack of owner education about these animals (17.2%), inability of natural living and expressing species specific behavior (13.8%), living in captivity (12.6%), inadequate knowledge about exotic animals in general (9.2%), and stress/inability to adapt to living conditions, inappropriate nutrition and inadequate number of professionals ex-perienced in treating exotic animals (10.5%). Keeping conditions of exotic pet mammals in pet shops and commercial breeding facilities were considered inappropriate by 70.5% and 51.6% of students, respectively.

Students neither agreed nor disagreed that pet rodents, rabbits or ferrets posed a threat to humans or other animals, and disagreed that they posed a threat to the environment, with no significant between-mammal differences (Table 3).

**Figure 1:**
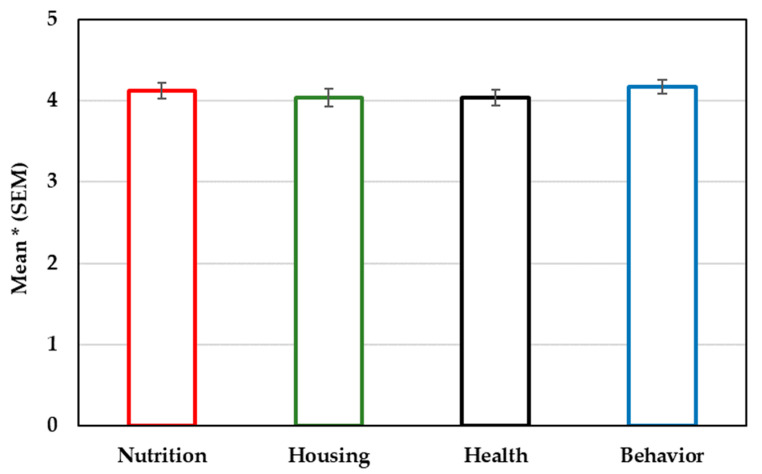
Responses of final-year veterinary students (*n* = 95) on their self-reported knowledge about exotic pet mammal nutrition, housing, health and behavior. * 1—completely disagree; 5—completely agree.

Regarding their self-reported knowledge on exotic pet mammals (mean ± SEM), students considered themselves to have appropriate knowledge about their nutrition (4.12 ± 0.10), housing (4.04 ± 0.11), health (4.04 ± 0.10) and behavior (4.17 ± 0.09), with no significant differences among these education areas (Figure 1). However, 84.2% of students thought the number of study subjects related to exotic pet animals to be inadequate.

There was no significant association of student gender, early environment, secondary education, study track chosen, prior owning or keeping pets with their responses to questions related to exotic pets. Student age was found to influence their perception of exotic pets, i.e., older students were significantly more inclined (*p* < 0.05) to report that exotic pet mammals posed a threat to humans and other animals, as well as to the environment, although being undecided about the issues (Table 4). Previous owning or keeping exotic pet animals showed a substantial association with student perception of these pets. Students with previous experience with exotic pets were significantly more aware (*p* < 0.05) of cognitive abilities and sentience of the pet mammals observed, as well as of the role of natural living for their welfare, but were significantly less likely (*p* < 0.05) to believe that these animals posed a threat to humans, other animals and the environment, disagreeing with the latter issues (Table 5).

## 4. Discussion

Students perceived rodents, rabbits and ferrets as animals capable of thinking and feeling emotions, considering biological functions, emotional states and natural living as the important components of the pet mammal welfare. There are a number of reasons for concern related to the welfare of these pets [1,2,25], as also believed by students. Our students called into question the welfare of all animals under study, with no significant differences among particular mammals, suggesting that they associated animal cognitive abilities and sentience with their welfare. There was no significant difference in student responses to any of the statements between rodents and rabbits, or between these animals and ferrets, with the exception of the statement on their suitability as pet animals. Students agreed that rodents and rabbits were suitable as pets, while being undecided whether this held true for ferrets. These findings could be explained by the students keeping and encountering rodents and rabbits as pets more frequently than ferrets.

Numerous studies point to inadequate owner level of information and knowledge of exotic pets and their needs [26,27,28], with owners frequently underestimating the issues related to keeping exotic pets [2]. Our students listed inappropriate accommodation and lack of respective education in the owners as the most relevant issues for the welfare of exotic pet mammals, with borderline agreement on the owners being properly informed on rodents and rabbits prior to getting them as pets, while being uncertain relative to ferrets, however, with no significant between-mammal differences.

The majority of students thought that exotic pet mammals were kept in inappropriate conditions in pet shops and breeder facilities. These results are consistent with previous reports [29,30,31].

As compared to previous studies on non-mammal pet animals [23,24], except for the birds, results of the present study suggest a higher student awareness of mammals as pet animals in terms of their welfare, and similar findings were recorded on the other issues involved, yielding no significant differences among particular mammals.

Students were uncertain whether pet mammals observed posed a threat to health and safety of humans and other animals. There are a number of diseases that can be transmitted from ferrets to humans, and veterinarians should be aware of these diseases [32,33]. Ferrets are the gold-standard animal model for influenza virus infection and can also carry and transmit COVID-19 to humans [34]. In this regard, veterinarians may be the first to diagnose emerging zoonotic diseases in ferrets, which puts them at a higher risk of exposure [35]. A threat posed by disease transmission from rodents and rabbits to humans is by no means less hazardous, including diseases such as salmonellosis, lymphocytic choriomeningitis, rat bite fever, hantaviruses, mpox, leptospirosis, ringworm, etc. [33,36,37]. Besides increasing the risk of disease onset, in particular transmission of new pathogens to humans, exotic pets are a potential source of disease for other pets and livestock [33,38]. Health issues can also imply allergies to exotic pets [39]. Moreover, ferrets can cause potentially dangerous injuries, with infants and small children being particulary vulnerable [40,41]. Animal inflicted injuries may also include rodents and rabbits [42]. Bites from these animals usually do not cause profound injuries but serve as an important mechanism of zoonotic disease transmission [33].

Students believed that none of the pet mammals under study posed a threat to the environment. In their study, D’Ovidio and Pirrone [43] demonstrated that one-fourth of surveyed owners of pet squirrels in Europe kept invasive alien species of EU concern (*S. carolinensis* and *T. sibiricus*), almost all of which were sexually intact, thus being a serious threat to biodiversity. Harris [44] reports that ferrets are under strictest legal regulations among all common exotic species of small mammals in the US. Being both potential rabies vectors and an invasive species, keeping ferrets is illegal in some US states and territories.

Considering that our students evaluated the environmental threat posed by pet rodents, rabbits or ferrets as lowest, we can presume that they were guided by the situation encountered in Croatia. Concerning other issues of concern related to threats that exotic pet mammals may pose to health and safety of humans and other animals, students must have failed to think about it thoroughly, however, the last-year veterinary students should be able to readily answer questions on the implications these animals may have for health of humans, as well as of other animals, comprehending the far-reaching consequences of their keeping as pets. On the other hand, students stated to have appropriate knowledge about nutrition, housing, health and behavior of these pets, with no significant differences among the areas of education observed; which was not the case in previous studies on exotic non-mammal pets [23,24]. Nevertheless, the majority of students considered that the number of study subjects involving exotic pets was inadequate, which is in line with the study conducted by Espinosa García-San Román et al. [21].

Studies show that many veterinarians lack both the knowledge about exotic pets and available resources in practice [28,45]. However, in the future, veterinarians can expect to encounter ever more such pets in their practice and should be ready to face this challenge [46]; the more so, increased engagement may increase their confidence in the field [47]. Rosenthal [48] believes that veterinary faculties should reconsider their curricula and rearrange their required and optional subjects. The needs of veterinarians having graduated with rather poor medical knowledge about exotic pets should be addressed by ensuring continuing education and professional development.

Considering socio-demographic parameters under study, student age and in particular previous experience with exotic pets were found to influence their perception of these pets. Older students were significantly more likely to believe that these animals were a threat to health and safety of humans. Although they were uncertain, the findings could be related to older students being married and/or having young children, previously shown to affect people’s practices and attitudes regarding zoonoses [49] and probably projecting it to the health and safety of other animals and the environment. The students having previously owned or kept exotic pets perceived cognitive abilities and sentience of these pets and the role of natural living for their welfare significantly better. These results confirm that previous experience with particular animals has a considerable effect on differences in human perception of these animals [50], as well as on the development of personal and professional values in veterinary students [51,52]. However, in comparison to other students, significantly more students having previous experience with exotic pets did not agree on these animals to pose a threat to humans, other animals and the environment. This might be explained by their having no adverse experience regarding these issues and pets owned or kept. Other socio-demographic parameters including study track chosen showed no significant association with student responses, suggesting their rooted perception and knowledge about these pets on the one hand and the need of extending education in exotic pet medicine on the other hand.

## 5. Conclusions

Results of the present study point to an adequate perception of final-year veterinary students of welfare issues in exotic pet mammals and that they considered themselves to have appropriate knowledge about nutrition, housing, health and behavior of these pets. However, other issues involving health and safety of humans and other animals, and environmental protection, require their additional attention. The veterinarians-to-be should be fully aware of these issues in order to be able and competent to properly inform the owners and keepers on the threats exotic pets may pose for humans, other animals, and eventually for the environment. Previous experience with exotic pets was found to substantially influence their perception, suggesting intensification of student interaction with these animals by upgrading veterinary curriculum in the field, and increasing the number of theoretical and practical classes. This especially applies to education about human health risk posed by these pets. The results obtained by the study can serve as a basis for further investigations into veterinary student perception and knowledge of these pets.

## Figures and Tables

**Table 1:** Socio-demographic profile of student sample (*n* = 95).

Parameter	% of Students	Parameter	% of Students
Gender		Early environment	
Male	25.3	Rural	28.4
Female	74.7	Urban	71.6
Age (yrs)		Secondary school	
<24	20.0	High school	94.7
≥24	80.0	Veterinary school	1.1
Prior ownership or keeping pets		Other	4.2
Yes	96.8	Study track chosen	
No	3.2	Pet animals	49.5
Prior ownership or keeping exotic pets		Farm animals and horses	21.1
Yes	53.7	Hygiene and technology of animal foodstuffs and veterinary public health	29.4
No	46.3		

**Table 2:** Responses of final-year veterinary students (*n* = 95) to statements referring to welfare issues in pet rodents, rabbits and ferrets.

Statement	Rodents	Rabbits	Ferrets
	Mean * (SEM)		
These mammals are capable of thinking	3.88 (0.10)	3.85 (0.11)	3.98 (0.11)
These mammals are capable of feeling emotions	3.88 (0.11)	3.91 (0.11)	3.82 (0.11)
Biological functions are important for their welfare	4.85 (0.06)	4.85 (0.06)	4.82 (0.07)
Emotional states are important for their welfare	4.10 (0.12)	4.08 (0.13)	4.10 (0.13)
Natural living is important for their welfare	4.78 (0.07)	4.79 (0.07)	4.76 (0.07)
These mammals are suitable as pets	3.91 ^a^ (0.10)	3.83 ^a^ (0.11)	3.40 ^b^ (0.13)
Owners are adequately informed prior to getting these pets	3.46 (0.13)	3.45 (0.13)	3.31 (0.14)
The welfare of these pet mammals is compromised	3.78 (0.10)	3.72 (0.10)	3.68 (0.11)

* 1—completely disagree; 5—completely agree; ^a,b^—different letters mark significantly different values (*p* < 0.05).

**Table 3:** Responses of final-year veterinary students (*n* = 95) to statements referring to threat that pet rodents, rabbits and ferrets pose to health and safety of humans and other animals, and environment.

Statement	Rodents	Rabbits	Ferrets
	Mean * (SEM)		
These pet mammals pose a threat to human health and safety	2.54 (0.13)	2.46 (0.12)	2.58 (0.13)
These pet mammals pose a threat to other animal health and safety	2.73 (0.13)	2.51 (0.13)	2.61 (0.13)
These pet mammals pose a threat to environment	2.32 (0.14)	2.26 (0.13)	2.25 (0.13)

* 1—completely disagree; 5—completely agree.

**Table 4:** Significant association of student age and responses to questionnaire statements.

Statement	Mammals	Age (yrs)	Mean *	SEM	F	*p*
These pet mammals pose a threat to human health and safety	Rabbits	<24	1.95	0.26	4.69	0.033
		≥24	2.59	0.13		
	Ferrets	<24	2.05	0.26	4.42	0.038
		≥24	2.71	0.14		
These pet mammals pose a threat to other animal health and safety	Rodents	<24	2.11	0.29	5.62	0.020
		≥24	2.88	0.15		
	Rabbits	<24	1.95	0.27	4.97	0.028
		≥24	2.65	0.14		
	Ferrets	<24	2.05	0.26	4.80	0.031
		≥24	2.75	0.15		
These pet mammals pose a threat to environment	Rabbits	<24	1.74	0.26	4.25	0.042
		≥24	2.40	0.15		

* 1—completely disagree; 5—completely agree.

**Table 5:** Significant association of student prior owning or keeping exotic pets (PrEP) and responses to questionnaire statements.

Statement	Mammals	PrEP	Mean *	SEM	F	*p*
These mammals are capable of thinking	Rodents	Yes	4.08	0.12	4.98	0.028
		No	3.66	0.15		
	Rabbits	Yes	4.16	0.12	10.56	0.002
		No	3.50	0.16		
	Ferrets	Yes	4.26	0.13	8.52	0.004
		No	3.66	0.16		
These mammals are capable of feeling emotions	Rodents	Yes	4.12	0.14	5.49	0.021
		No	3.61	0.17		
	Rabbits	Yes	4.18	0.13	7.68	0.007
		No	3.59	0.17		
	Ferrets	Yes	4.04	0.14	4.75	0.032
		No	3.57	0.16		
Natural living is important for their welfare	Rabbits	Yes	4.92	0.05	4.48	0.037
		No	4.64	0.13		
	Ferrets	Yes	4.92	0.05	6.46	0.013
		No	4.57	0.14		
These pet mammals pose a threat to human health and safety	Rodents	Yes	2.20	0.14	8.69	0.004
		No	2.93	0.21		
	Rabbits	Yes	2.14	0.14	9.07	0.003
		No	2.84	0.20		
	Ferrets	Yes	2.26	0.16	8.05	0.006
		No	2.96	0.20		
These pet mammals pose a threat to other animal health and safety	Rodents	Yes	2.37	0.16	8.71	0.004
		No	3.14	0.21		
	Rabbits	Yes	2.18	0.15	8.27	0.005
		No	2.89	0.21		
	Ferrets	Yes	2.29	0.16	7.35	0.008
		No	2.98	0.20		
These pet mammals pose a threat to environment	Rodents	Yes	1.88	0.14	13.01	0.001
		No	2.82	0.23		
	Rabbits	Yes	1.86	0.14	12.37	0.001
		No	2.73	0.21		
	Ferrets	Yes	1.82	0.14	14.32	<0.001
		No	2.75	0.21		

* 1—completely disagree; 5—completely agree.

## Data Availability

The original contributions presented in the study are included in the article and Supplementary Materials; further inquiries can be directed to the corresponding authors.

## Supplementary Materials

The following supporting information can be downloaded at: https://www.mdpi.com/article/10.3390/vetsci12030235/s1, Questionnaire survey as presented in the article.
