# Supplementary material for: Perception and Knowledge of Final-Year Veterinary Students About Exotic Pet Mammals
Source: Vet Sci. 2025 Mar 3;12(3):235. doi: 10.3390/vetsci12030235 (PMC11946023; doi:10.3390/vetsci12030235)
Supplement: Supplementary file 1 [file vetsci-12-00235-s001.zip › vetsci-3399966-supplementary.pdf]

Survey questionnaire as presented in the article.

1. Gender: a) Male b) Female
2. Age (years): ___________
3. Early environment: a) Rural b) Urban
4. Secondary school: a) High school b) Veterinary school c) Other ___________
5. Have you owned or kept pet animals? a) Yes b) No
6. Have you owned or kept exotic pet animals (i.e., any animals other than dogs and cats)? a) Yes b) No
7. If your answer to the above question is Yes, name the animal(s).

---

8. Chosen study track: a) Pet animals b) Farm animals and horses c) Hygiene and technology of animal foodstuffs and veterinary public health
9. Please indicate your level of agreement/disagreement (5–completely agree, 4–agree, 3–neither agree nor disagree, 2–disagree, 1–completely disagree) with the statement that the following mammals are capable of thinking:

Rodents	5	4	3	2	1
Rabbits (*Oryctolagus cuniculus*)	5	4	3	2	1
Ferrets (*Mustela putorius furo*)	5	4	3	2	1
Non-human primates	5	4	3	2	1

10. Please indicate your level of agreement/disagreement (5–completely agree, 4–agree, 3–neither agree nor disagree, 2–disagree, 1–completely disagree) with the statement that the following mammals are capable of feeling emotions:

Rodents	5	4	3	2	1
Rabbits	5	4	3	2	1
Ferrets	5	4	3	2	1
Non-human primates	5	4	3	2	1

11. Please indicate your level of agreement/disagreement (5–completely agree, 4–agree, 3–neither agree nor disagree, 2–disagree, 1–completely disagree) with the following statements related to pet mammals:

	Rodents	Rabbits	Ferrets	Non-human primates
Biological functions are important for their welfare				
Emotional states are important for their welfare				
Natural living is important for their welfare				

12. Please indicate your level of agreement/disagreement (5–completely agree, 4–agree, 3–neither agree nor disagree, 2–disagree, 1–completely disagree) with the statement that the following mammals are suitable as pets:

Rodents	5	4	3	2	1
Rabbits	5	4	3	2	1
Ferrets	5	4	3	2	1
Non-human primates	5	4	3	2	1

13. Please indicate your level of agreement/disagreement (5–completely agree, 4–agree, 3–neither agree nor disagree, 2–disagree, 1–completely disagree) with the statement that owners are adequately informed about the following mammals and their needs prior to getting them as pets:

Rodents	5	4	3	2	1
Rabbits	5	4	3	2	1
Ferrets	5	4	3	2	1
Non-human primates	5	4	3	2	1

14. Please indicate your level of agreement/disagreement (5–completely agree, 4–agree, 3–neither agree nor disagree, 2–disagree, 1–completely disagree) with the statement that welfare of the following pet mammals is compromised:

Rodents	5	4	3	2	1
Rabbits	5	4	3	2	1
Ferrets	5	4	3	2	1
Non-human primates	5	4	3	2	1

15. According to your opinion, state the most important issue for welfare of exotic pet mammals.

---

16. Do you consider that exotic pet mammals have appropriate keeping conditions in pet shops? a) Yes b) No c) I do not know

17. Do you consider that exotic pet mammals have appropriate keeping conditions in commercial breeding facilities? a) Yes b) No c) I do not know

18. Please indicate your level of agreement/disagreement (5–completely agree, 4–agree, 3–neither agree nor disagree, 2–disagree, 1–completely disagree) with the statements that the following pet mammals are threat to:

	Rodents	Rabbits	Ferrets	Non-human primates
Health and safety of humans				
Health and safety of other animals				
Environment				

19. Please indicate your level of agreement/disagreement (5–completely agree, 4–agree, 3–neither agree nor disagree, 2–disagree, 1–completely disagree) with the following statements related to pet mammals:

I have appropriate knowledge about their nutrition	5	4	3	2	1
I have appropriate knowledge about their housing	5	4	3	2	1
I have appropriate knowledge about their health	5	4	3	2	1
I have appropriate knowledge about their behavior	5	4	3	2	1

20. Do you think there is an adequate number of subjects related to exotic pets during the study? a) Yes b) No c) I do not know

21. When you finish your study, would you like to work exclusively with exotic pets? a) Yes b) No c) I do not know
